# Editorial

**Published:** 1847-07

**Authors:** 


### THE MEDICAL EXAMINER.

PHILADELPHIA, JULY, 1847.

### SHIP FEVER.

In our last we spoke of the vast number of emigrants arriving at the different sea-ports of the United States, in a condition of the utmost destitution, and in many instances laboring under ship-fever. This state of things continues, and, in fact, appears to be constantly on the increase ; nor does there seem to be any hope that it will be otherwise until the famine in Ireland disappears, or more efficient means are adopted to prevent so many persons from being crowded in the vessels that convey them hither, as well as to provide better provision for them on the passage.

It is not only in the Atlantic cities that cases of this fever are found, but wherever the unfortunate emigrants seek a home speedily after their arrival on the continent of America, from the Mississippi to the St. Lawrence. As the voyage from Ireland to the Northern ports is much shorter, of course the greatest number proceed thither, and consequently there the evils most abound. Some idea may be formed of the dreadful mortality among these poor people, who arrive in the St. Lawrence, from the following statement, which we cut from one of the newspapers, and in a smaller proportion, perhaps, among those who arrive at the ports of the United States :

" *Ship and Typhus Fever.*—A letter from Dr. Douglass at the Quarantine Station, Gross Isle, dated June 18, received in Montreal, gives some idea of the melancholy condition of vessels quarantined there.

The Pursuit, Spencer, from Liverpool—the master, mate, and all the men, save one, sick in hospital—was obliged to send hands from the shore, to remove his sick and dead.

The Lotus, Watson, from Liverpool—has had some of his sick removed to Hospital—expects to land the rest of the sick in a day or two—he had 70 deaths—12 since her arrival.

The Rose, M'Kinlay, from ———, has nearly 100 sick—lost 14 the day of his arrival, and 7 the day after—total deaths nearly 80.

The Lady Flora Hastings, from Cork—passengers landed, except the sick—72—who are still on board—has buried 60.

The ship Sabraon, Wilson, from Liverpool—has about 60 sick—buried 35—has a medical man on board, who attends to the sick.

The Jessie Gorman, from Limerick—sick 45, still on board—mate, and ten of the crew ill—buried 30 of his passengers."

The disease appears to be the old ship, jail or hospital fever, modified in different individuals, so as to constitute either *typhus* or *typhoid* fever, according to the notions of different writers. In all instances it may be regarded as adynamic in its character; sometimes with lesions of the intestinal glands, and in other cases without such lesions, but presenting black tongue, petechiæ, great stupor and prostration, &c. From the number of cases occurring, and the fact that in a few instances the attendants on the sick have contracted and even died of the disease, some alarm has been experienced from the apprehension that it may spread beyond its present limits and become general among the people. We do not, however, participate in this apprehension, nor do we believe that it is entertained by any considerable portion of the physicians of this or any other place where the disease has appeared. That it is contagious, under peculiar circumstances of exposure, and that a few persons have fallen victims to it under such circumstances, may be admitted, and is admitted; but the smallness of the number out of all who have been so situated, shows how rarely it is contracted by those who are not predisposed to it by the melancholy circumstances of the chief sufferers, whilst there are no instances of its occurrence beyond the immediate sphere of infection. These circumstances show no tendency in the disease to spread, and indicate anything but a state of the atmosphere favourable to its propagation. Had the disease appeared among us in cold weather instead of the summer season, its effects upon those who are obliged to attend upon the sick in our almshouses and other places of public charity, would probably have been deplorable.

UNIVERSITY OF PENNSYLVANIA.

Professor Robert Hare, who has occupied the Chair of Chemistry in this time-honoured School of Medicine nearly thirty years, has resigned his professorship, and we understand that at the meeting of the Trustees at which his resignation was accepted, he was unanimously elected Emeritus Professor of Chemistry—a proper acknowledgement of distinguished abilities and long and faithful services. No successor has yet been appointed.

---

HAMPDEN SYDNEY COLLEGE.

In our last we announced the decease of Dr. Warner, Professor of Surgery in this institution. From the following notice, which we copy from the U. S. Gazette, it will be seen that a successor, of reputation, has already been appointed.

"Dr. James L. Cabell, now Professor of Anatomy and Surgery in the University of Virginia, has been selected, and has consented, to take charge of the professorship in the Richmond Medical College, recently made vacant by the death of the late Dr. Augustus L. Warner. Dr. Cabell is now widely known as a medical instructor, and will prove a valuable accession both to the college and the city."

---

BUFFALO UNIVERSITY.

The first Annual Commencement of the Medical Department of the Buffalo University, took place on the 16th inst. The degree of Docter of Medicine was conferred on seventeen graduates.

---

LA LANCETTE CANADIENNE.

But a few short months since, we chronicled the advent of this spirited little French organ of medical science, and now, alas! it is numbered with those that have been! The number which appeared on the 15th ultimo, contained the valedictory of its able editor, Dr. Leprohon.

---

NEW YORK MEDICAL AND SURGICAL REPORTER.

The Boston Medical and Surgical Journal of the 23d ultimo, announces the discontinuance of its weekly contemporary, and takes occasion to say that "the Boston Journal is again alone in this country as a weekly visitant to the medical profession."

Beware, brother Smith! Listen to the Bard—

"This is the state of man:—to day he puts forth
The tender leaves of hope, to-morrow blossoms,
And bears his blushing honours thick upon him;
The third day comes a frost—a killing frost!"

### ARMY SURGEONS.

The Army Medical Board, which was recently convened in New York, for the purpose of examining candidates for appointment to the Medical Staff of the regular army, has approved the following gentlemen as being properly qualified:

Nicholas L. Campbell, New York.
Samuel L. Barbour, Georgia.
George Edward Cooper, Pennsylvania.
Ebenezer Swift, Ohio.
John. S. Battee, Maryland.
Glover Perin, Ohio.
P. G. Stuyvesant Ten Broeck, New York.
John Campbell, New York.
John E. Summers, Virginia.
Charles A. Smith, Virginia.
Washington M. Ryer, New York.

Surgeon John B. Wells was also examined for promotion to that grade, and was fully approved by the same board.

---

### DEATH OF LISFRANC.

The late foreign Journals announce the death, at Paris, of this celebrated surgeon. The following account is from a letter written at Paris by Dr. F. W. Fisher, to his uncle at Boston, and published in the Boston Journal of the 9th of June:

"One of the most noted of the French surgeons has paid his tribute to nature. Lisfranc is dead! After four weeks of suffering from pseudo-membranous croup (*angine couenneuse*) he fell a victim to that relentless malady. He requested that tracheotomy might be performed for his relief; but that operation was objected to, as false membranes were already formed in the pharynx, œsophagus, larynx, bronchi, &c. He was aware of his situation, and said that he would not shrink before death *if his work on operative medicine were completed!* This remark of the dying Lisfranc is characteristic—not of Lisfranc alone, but of the French savans generally. It has often amused me to notice how jealous the French professors are of their scientific brethren, and how anxious they are to obtain professional notoriety. Lisfranc had become an old man, and yet he had all the feelings of youthful ambition. He had served as surgeon in the armies of Napoleon, and had been honorably noticed by that great man, and yet on the day of his death his craving for increased honour and glory seemed to be as imperious as ever. Lisfranc seems to have been a greater favorite with his countrymen than with foreign physicians and students. As a teacher he was much followed, and as a surgeon he deservedly held a high rank. He was a man of strong and violent prejudices—and never failed in exhibiting them in his lectures, whenever he spoke of those whom he considered as rivals. Dupuytren and Velpeau were the peculiar recipients of his censures—and he oftentimes spoke of these great men with a severity and an eloquence which must have characterized the age of the French revolution. All those who were familiar with the social character of Lisfranc, say that he was a warm-hearted man—that he was ever a friend of the poor, and that his services and his purse were ever at the call of suffering humanity."

This brief description of the able but eccentric Frenchman, is in accordance with a lively sketch of him published in this journal some years ago, from the pen of the then editor, Dr. Clymer, and to which our attention has been directed. Some of our readers may remember having read it at the time, but to most of them the language as well as the facts will be new, and as every one must feel interested in the personal character and peculiarities of one who has occupied so prominent a position in the eyes of the profession, the republication of our predecessor's remarks at the present time will not be deemed out of place.

"Few medical visitors to the French metropolis will readily forget the distinguished surgeon of La Pitié. The position which he has so long occupied as a clinical professor, the great reputation he has so worthily obtained, and the respect with which his professional opinions are received, render him an object of early interest and attention. His lofty stature, stately gait, and robust form ; his large and regular features, his deep set, restless and piercing eye, overshadowed by a heavy brow ; his ample forehead, with its few grey locks, surmounted by the old cap, and his pale and haggard expression, all make an appearance eminently striking and not easily forgotten. No man has been more misrepresented than M. Lisfranc. An unfortunate temperament has done much toward injuring his prospects ; and to disappointment, acting on a natural infirmity of disposition, may, perhaps, be attributed that eccentricity of character and impetuosity of temper, which are usually associated with his name. His capacity is undoubtedly of an exalted order ; surpassed, if equalled, by that of none of his rivals. A quick and vigorous understanding, a lively imagination, a clear and sure judgment, a varied and solid erudition, a persevering industry, and undaunted professional ardour, are his claims to a reputation laboriously and honestly obtained. With these intellectual powers are unhappily combined an aceticism of temper, a violence of prejudice, an impetuosity of feeling, and a selfish spirit, which betray him into the constant indulgence of violent personal invective, to the utter disregard and contempt of the feelings and claims of others. As a lecturer he is full of useful information, which he conveys in a clear, vigorous and energetic style, almost too theatrical to be impressive, and often offensive from self-sufficiency and importance. A consciousness of manifest superiority, and a determined inclination to assert and maintain it, lead him to the constant indulgence of his sarcastic humour, and his sallies, indiscriminately directed, are as unmeasured as in ge..eral unmerited. Frequently in the midst of a lecture of intense interest he will abruptly stop, his whole frame becoming convulsed, and, without apparent provocation, pour forth, in stentorian voice, a passionate and boisterous philippic on the devoted head of a *confrère*, garnished with admonitory expletives to the neighbouring internes. The fit over, he resumes his discourse, and again interests and delights his class. From his pupils he exacts an entire submission and passive obedience to his whims and caprices, any defection being visited by gross personal abuse, publicly administered. His manner to his patients is usually uncouth and repulsive, he frequently during his morning visits abandoning himself to sudden transports of rage; and in the course of a painful and tedious operation, he commonly exhausts several times his juratory vocabulary. During his life time, Dupuytren was the especial object of Lisfranc's hatred and abuse; since his death he has canonized him and his doctrines, and Velpeau has become the subject of his daily vituperation and invective."

Lisfranc was born the 12th of April, 1787, and died on the 12th of May last. On the 14th his remains were followed to the grave by an immense concourse of physicians and medical students.

